# Influence of nutritional status on patient-reported outcomes after surgical treatment for chronic venous disease: a comparative study between endovenous laser ablation and combined crossectomy, stripping, and sclerotherapy

**DOI:** 10.1007/s00423-026-04043-0

**Published:** 2026-04-18

**Authors:** Vinicius Nicolao Capacia, Raquel Bragança Soares Capacia, Ana Paula Ribeiro, Elias Jirjoss Ilias, Patrícia Colombo-Souza

**Affiliations:** 1 Postgraduate Program in Health Science, Santo Amaro University, 340 – Jardim das Imbuias, São Paulo, 04829-300 Brazil; 2 Santa Casa de São Paulo School of Medical Sciences, São Paulo, Brazil

**Keywords:** Chronic venous disease, Nutritional status, Obesity, Quality of life, Endovenous laser ablation, Sclerotherapy

## Abstract

**Purpose:**

This study investigated the influence of nutritional status on patient-reported outcomes after surgical treatment for chronic venous disease (CVD). It aimed to compare improvements in symptom severity (VEINES-Sym) and quality of life (VEINES-QOL) between endovenous laser ablation (EVLA) and combined crossectomy, stripping, and sclerotherapy (CEEE), with analysis stratified by nutritional status.

**Methods:**

In a prospective cohort study of 157 patients (95 CEEE, 62 EVLA), nutritional status was categorized by body mass index as eutrophic, overweight, or obese. VEINES-Sym and VEINES-QOL scores were collected preoperatively and 30–60 days postoperatively. Statistical analysis used paired t-tests for within-group comparisons, the Kruskal-Wallis test across nutritional strata, and independent t-tests to compare surgical techniques.

**Results:**

Both techniques produced significant improvements (*p* < 0.05) in VEINES-Sym and VEINES-QOL scores across all nutritional categories. Preoperatively, patients with obesity had significantly worse baseline scores. Postoperatively, these differences were no longer significant (*p* > 0.05), with all groups achieving comparable scores. The magnitude of symptom improvement was similar for both techniques. However, postoperative VEINES-QOL scores were significantly higher in the EVLA group (94.7 ± 9.9) than in the CEEE group (90.5 ± 13.7; *p* = 0.015).

**Conclusion:**

Although poorer nutritional status is associated with greater preoperative symptom severity, it does not diminish the significant clinical benefit of surgical intervention. Both CEEE and EVLA effectively improve symptoms and quality of life, enabling all patient groups to attain similar postoperative outcomes.

## Introduction

Chronic venous disease (CVD), with its manifestations ranging from telangiectasias to venous ulcers, is a condition of high prevalence and global impact [1,2]. In Brazil, estimates indicate that the disease affects more than 30% of the adult population, generating high costs for health systems and damaging individuals’ quality of life and productivity [3]. Among its several established risk factors - such as age, female sex, family history, and prolonged orthostatism - obesity stands out as one of the main modifiable elements [4]. Excess weight increases intra-abdominal and venous pressure in the lower limbs, promoting valvular dysfunction and reflux, and aggravating venous hypertension characteristic of CVD [5,6].

The management of symptomatic saphenous vein insufficiency, one of the pillars of CVD, evolved with the advent of minimally invasive techniques [4,7]. Endovenous laser ablation (EVLA) [8] has established itself as an effective and safe alternative to conventional surgery (CEEE - crossectomy, estripping (stripping), and excision of extended/collateral veins) [9], usually presenting lower morbidity and faster recovery. The evaluation of the success of these treatments, however, transcends the anatomical parameters of venous occlusion, incorporating, crucially, the measurement of patient-reported quality of life [10,11]. For this, validated instruments such as the VEINES-QOL/Sym questionnaire have become essential tools to capture improvement of symptoms and postoperative well-being [12,13].

Although obesity is a well-known risk factor for the development of CVD, its specific influence on postoperative outcomes remains a less explored area [14]. It is plausible to assume that the nutritional status - classified in eutrophication, overweight, and obesity - may act as a modifier of the surgical outcome [15]. Differences in inflammatory response, microcirculation, healing process, and even associated comorbidities could modulate the effectiveness of different techniques unevenly [16]. While EVLA is a thermal ablation dependent on the local tissue response, CEEE involves more extensive surgical trauma; nutritional status may intervene distinctly in the recovery of each of them [11].

Given the above, this study aimed to compare, in a stratified way by nutritional status, the evolution of symptom scores (VEINES-Sym) and quality of life (VEINES-QOL) in the pre- and postoperative. We seek to determine whether the nutritional status is a decisive factor for the choice of technique, aiming at a truly personalized vascular surgical practice.

## Materials and methods

### Study design and context

A prospective, non-randomized cohort study was conducted according to the STROBE guidelines. Data collection took place between January 2022 and August 2025 in two centers: a public hospital (Santa Casa de Guaratinguetá) and a private clinic (HumaniClin), representing the public and private health systems of the state of São Paulo, Brazil.

### Participants

The original population consisted of all adult patients (18 years) undergoing surgical treatment for chronic venous disease (CVD) in participating centers during the study period. We included patients with DVC (CEAP 2–6 classification) and insufficiency of at least one segment of the saphenous vein magna or parva (reflux 0.5 s to ecodoppler), submitted to correction with EVLA or CEEE, who completed the quality of life questionnaire in a valid way (> 50% of items) preoperatively and postoperatively (30–60 days).

Patients submitted to other surgical techniques (e.g., radiofrequency, conventional safenectomy), with CEAP 1 (only telangiectasias) or with incomplete follow-up data, were excluded from the study.

### Sample size

The sample size was defined for convenience, including all eligible patients during the recruitment window. A post-hoc power analysis was performed based on the primary outcomes.

### Selection of sample

**Fig. 1:**
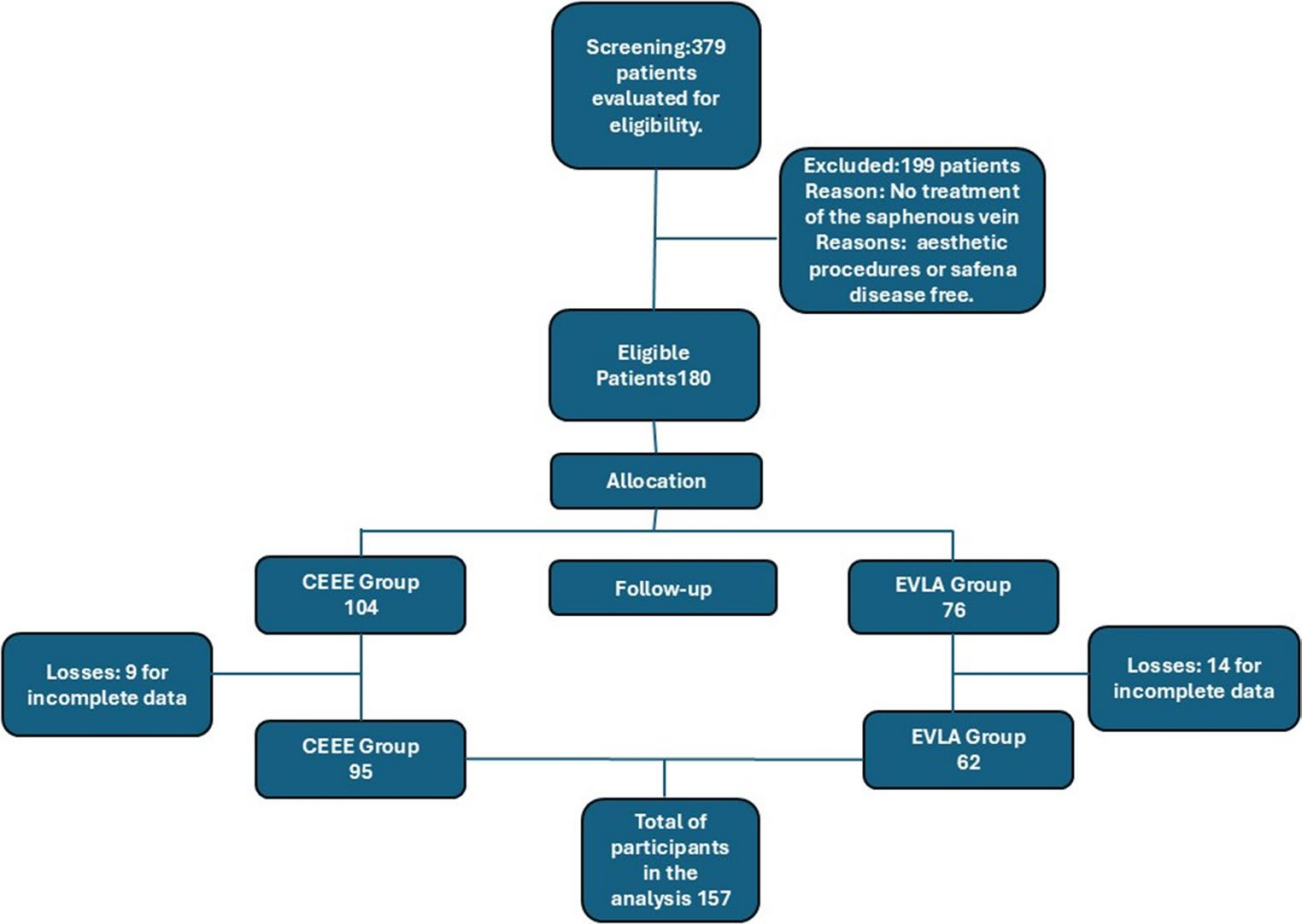
Flow diagram of patient recruitment and follow-up

The recruitment of participants followed the flow shown in Fig. 1. Initially, 379 patients were screened for eligibility. Of these, 199 were excluded because they had not performed saphenous vein treatment (aesthetic procedures). Of the 180 eligible patients, 104 were allocated to the CEEE group and 76 to the EVLA group. After applying the post-allocation exclusion criteria (incomplete data), the final cohort for analysis was composed of 157 patients (95 in the CEEE group and 62 in the EVLA group).

### Variables

Exposure variables: surgical technique (EVLA or CEEE) and nutritional status. The nutritional status was categorized by Body Mass Index (BMI) in eutrophic (BMI < 25 kg/m ^2^), overweight (BMI 25–29.9 kg/m ^2)^, and obesity (BMI 30 kg/m ^2^).

Outcome Variables: The primary outcomes were the symptoms and quality of life scores, measured by the validated VEINES-Sym and VEINES-QOL questionnaires, applied in preoperative and postoperative (30–60 days), in which higher scores indicate better results.

Potential confounding variables: Data on age, sex, CEAP classification, and comorbidities (e.g., hypertension, diabetes) were collected to characterize the sample and evaluate comparability between groups.

### Data sources and measurement

Data were extracted from electronic and physical medical records, as well as from the surgical databases of the two centers. The venous insufficiency was confirmed by venous ecodoppler performed by medical specialists (radiologists or vascular surgeons). The VEINES-QOL/Sym questionnaires were applied in a standardized way: by self-filling in the private clinic and by direct interview in the public hospital, to mitigate the literacy bias.

### Bias

To minimize selection bias, all consecutive patients who met the criteria throughout the study period were included. The information bias was mitigated by the use of validated questionnaires and standardized data collection.

### Statistical analysis

The normality of the data was evaluated by the Shapiro-Wilk test. Continuous data is presented as mean, standard deviation, and categorical data as frequencies (n, %). To compare the VEINES scores in the pre- and postoperative, within each group (EVLA-Eutrophication pre- and post), we used the paired Student t test.

To compare the scores between the nutritional status groups, in the same technique (VEINES-Sym postoperative: Eutrophication, Overweight and Obesity in the EVLA group), we used the Kruskal-Wallis Test. To compare the outcomes between surgical techniques (EVLA vs. CEEE), we used the Student t test for independent samples or the Mann-Whitney test.

Confidence intervals of 95% (CI 95%) were calculated for the relevant differences. The level of significance adopted was *p* < 0.05. The analyses were performed in the software BioEstat 5.3.

### Surgical methods (interventions)

Endovenous Laser Ablation (EVLA): Performed with 1470 nm diode laser (ORlight INNOVA CX), flat fiber, with endovenous linear energy density (LEED) between 50 and 90 J/cm. Associated with phlebectomy and sclerotherapy with polydocanol foam 1%.

Phlebectomy surgery combined with sclerosis with polydocanol foam, ecoguided. - CEEE: Performed with polydocanol foam (1% or 3%, depending on venous size), produced by the Tessari technique, under ultrasound guidance, associated with phlebectomy and perforating ligation.

### Ethical aspects

The study was approved by the Research Ethics Committee of the University of Santo Amaro (CEP-Unisa, Opinion no 7.408.144), in accordance with Resolution CNS 466/2012. Free and informed consent was obtained from all participants.

## Results

A total of 157 patients were included in the final analysis: 95 submitted to CEEE and 62 to EVLA. The sociodemographic and clinical profile of the total sample, as well as the comparison between groups, are detailed in Table 1. The groups were comparable in terms of age, nutritional status, prevalence of comorbidities, and severity of disease, according to the CEAP classification (all with *p* > 0.05). However, significant differences were observed in the distribution by sex (78.9% women in the CEEE group and 69.4% in the EVLA group; *p* < 0.0001) and in the type of occupation, with a significantly higher proportion of patients with manual occupation in the CEEE group (47.2% vs. 22.6% in the EVLA group; *p* = 0.012).

**Table 1:** Comparative analysis of sociodemographic characteristics, comorbidities, and CEAP classification among patients submitted to CEEE and EVLA

Sociodemographic characteristics	Surgical methods				Total	*p*-value*
	CEEE		EVLA			
	*N*	%	*n*	%		
Sex						
Female	75	78.9	43	69.4	118	< 0.0001^a^* CEEE> Female
Male	20	21.1	19	30.6	39	
Total	95	100.0	62	100.0	157	
Nutritional status						
eutrophic	30	31.5	29	45.2	57	0.096^a^ N.S.
overweight	19	19.6	11	14.5	27	
obesity	46	48.9	22	32.3	65	
Total	95	100.0	62	100.0	149	
Age						
Age in years	52.6 ± 11.3 (29–83 years) *n* = 95		53.5 ± 12.4 (18–76 years) *n* = 62		157	0.3189^b^ N.S.
Occupation						
Handyman (H)	43	47.2	12	22.6	55	0.012^a^* EEE > H EVLA > A
Administrative (A)	26	28.6	24	45.3	50	
Undetermined (U)	22	24.2	17	32.1	39	
Total	91	100.0	53	100.0	144	
Comorbidades						
hypertension	35	36.8	19	30.8	54	0.2248^a^ N.S.
diabetes mellitus	20	21.1	6	9.7	26	
hypothyroidism	7	7.3	6	9.7	13	
mental disorders	6	6.3	8	12.9	14	
vdyslipidemia	8	8.5	7	11.2	15	
Total	76	100.0	46	100.0	122	
CEAP (clinical, etiology, anatomy, pathophysiolog)						
2	22	23.1	17	27.4	39	0.6096^a^ N.S.
3	51	53.7	29	46.7	80	
4	9	9.5	10	16.2	19	
5	3	3.2	2	3.3	5	
6	10	10.5	4	6.4	14	
Total	95	100.0	62	100.0	157	

*NS* Not significant (*p* > 0.05); * *p* < 0.05Data presented as n (%) or mean ± standard deviation [a] P value from Chi-Square test [b] P value from Student’s t-test for independent samples

The analysis of the primary outcome, stratified by surgical technique and nutritional status, is presented in Table 2. Both procedures promoted a statistically significant and clinically relevant improvement in symptom (VEINES-Sym) and quality of life (VEINES-QOL) scores from pre to postoperative in all strata of nutritional status (all p values < 0.05). Notably, in the preoperative period, patients with obesity, regardless of technique, presented the worst basal scores for VEINES-Sym and VEINES-QOL.

**Table 2:** Comparison of symptom scores (VEINES-Sym) and quality of life (VEINES-QOL) in pre- and postoperative, stratified by surgical technique (EVLA vs. CEEE) and nutritional status

Nutritional status	Surgical methods	Period	VEINES-Sym (score)	VEINES-QOL (score)	*p*-value*	CI 95%
eutrophic	EVLA	Pre (*n* = 29)	36.32 ± 8.29	81.7 ± 14.1	< 0.001*	-12.85 a -5.78
		Post (*n* = 29)	45.64 ± 5.10	96.29 ± 10.6	< 0.0001*	-20.48 a -8.54
	CEEE	Pre (*n* = 30)	35.41 ± 6.69	77.8 ± 13.34	< 0.001*	-14.02 a -8.18
		Post (*n* = 30)	46.51 ± 5.14	93.55 ± 12.27	< 0.0001*	-21.04 a -10.47
overweight	EVLA	Pre (*n* = 11)	38.7 ± 5.4	81.5 ± 10.9	< 0.0027*	-10.55 a -2.55
		Post (*n* = 11)	45.3 ± 5.12	94.5 ± 9.51	< 0.0031*	-21.13 a -4.86
	CEEE	Pre (n19)	35.83 ± 7.17	44.4 ± 6.03	< 0.0001*	-12.30 a -4.92
		Post (*n* = 19)	76.11 ± 14.6	88.5 ± 15.6	< 0.0003*	-18.62 a -6.26
obesity	EVLA	Pre (*n* = 22)	29.5 ± 8.51	70.31 ± 16.6	< 0.001*	-18.05 a -9.20
		Post (*n* = 22)	43.1 ± 5.49	93.4 ± 10.29	< 0.0001*	-31.26 a -14.94
	CEEE	Pre (*n* = 46)	30.53 ± 7.22	44.6 ± 6.61	< 0.001*	-16.13 a -12.13
		Post (*n* = 46)	67.86 ± 14.41	89.86 ± 14.18	< 0.0001*	-26.36 a -17.63

*Paired Student’s t-test for comparison between preoperative and postoperative periods within each group. Data presented as mean ± standard deviation. *CI *confidence interval.*p < 0.05 was considered statistically significant*

To investigate whether the nutritional status influenced the magnitude of the improvement, we compared the scores between the groups of nutritional status for each technique, as shown in Table 3. In the preoperative period, a significant difference was observed in the scores between eutrophic, overweight, and obese patients for both techniques (*p* < 0.05 for VEINES-Sym and VEINES-QOL). This initial heterogeneity, however, was completely abolished in the postoperative period, since there were no significant differences in scores between nutritional states after surgical intervention (*p* > 0.05 in all postoperative comparisons).

**Table 3:** Comparison of symptom scores (VEINES-Sym) and quality of life (VEINES-QOL) in the pre- and postoperative period between nutritional status groups, according to the surgical technique

VEINES questionary	Surgical methods	Period	eutrophic (*n* = 29)	overweight (*n* = 11)	obese (*n* = 22)	*p*-value*
VEINES-Sym	EVLA	Pré	36.32 ± 8.29	38.77 ± 5.40	29.52 ± 8.51	0.007*
		Pós	45.64 ± 5.10	45.33 ± 5.12	43.10 ± 5.49	0.224
VEINES-QOL	EVLA	Pré	81.7 ± 14.1	81.5 ± 10.9	70.31 ± 16.6	0.045*
		Pós	96.29 ± 10.6	94.5 ± 9.51	93.4 ± 10.29	0.657
VEINES Questionary	Surgical Methods	Period	eutrophic (*n* = 29)	overweight (*n* = 11)	obese (*n* = 22)	p-value*
VEINES-Sym	CEEE	Pré	35.41 ± 6.69	35.83 ± 7.17	30.53 ± 7.22	0.006*
		Pós	46.51 ± 5.14	44.4 ± 6.03	44.6 ± 6.61	0.319
VEINES-QOL	CEEE	Pré	77.8 ± 13.34	76.11 ± 14.6	67.86 ± 14.41	0.015*
		Pós	93.55 ± 12.27	88.5 ± 15.6	89.86 ± 14.18	0.145

*Data presented as mean ± standard deviation.* *p-value refers to the Kruskal-Wallis test for comparison among the three nutritional status groups (Eutrophic, Overweight, and Obese) within each surgical technique and period. A p-value < 0.05 was considered statistically significant.*

Table 4 shows the direct comparison of scores between the two surgical techniques. In the preoperative period, the groups were comparable for VEINES-Sym (*p* = 0.219), but there was a small, albeit significant, difference in VEINES-QOL (*p* = 0.018). In the postoperative period, both techniques showed substantial improvements, with no significant difference in symptom score (VEINES-Sym, *p* = 0.339). However, the EVLA group obtained a mean quality of life score (VEINES-QOL) significantly higher than that of the CEEE group in the postoperative period (94.7 9.94 vs. 90.5 13.7; *p* = 0.015).

**Table 4:** Comparison of VEINES-Sym and VEINES-QOL pre- and postoperative scores in patients submitted to EVLA versus CEEE

VEINES Questionary	Period	EVLA (*n* = 62)	CEEE (*n* = 95)	Diference (CI 95%)	*p*-value*
VEINES-Sym (score)	Pre	34.37 ± 8.38	33.15 ± 7.35	− 3.1 a 1.27	0.219
	Post	44.72 ± 5.19	45.1 ± 6.05	-1.52 a 2.16	0.339
VEINES-QOL (score)	Pre	77.7 ± 14.6	76.7 ± 14.5	-9.08 a -0.37	0.018*
	Post	94.7 ± 9.94	90.5 ± 13.7	-7.98 a − 0.357	0.015*

*Data presented as mean ± standard deviation; p-value refers to Student’s t-test for independent samples. *CI *confidence interval.*p < 0.05 was considered statistically significant*

## Discussion

Our results revealed a distinct sociodemographic profile among the groups, with the CEEE group showing a significantly higher proportion of women and patients with physical occupations (Table 1). The higher prevalence of females may reflect, in addition to the known higher incidence of CVD in women, a greater concern with aesthetic appearance and search for treatment, a phenomenon widely documented in the literature [4–6]. In addition, the association between the CEEE technique and the manual occupation is an indirect marker of the socioeconomic context of this group, mostly attended by the SUS. Patients from less privileged social classes are often inserted in occupations that involve prolonged orthostatism or physical effort, known risk factors for the worsening of venous symptoms [17]. This occupational overload, added to a possible more limited access to preventive care, may have contributed to the progression of the disease in this cohort, which justifies the search for surgical treatment.

The most frequently observed nutritional status in our sample was obesity, which corroborates its role as a significant risk factor for CVD. The pathophysiological mechanisms are multifactorial, including increased intra-abdominal pressure, which hinders venous return, and a chronic inflammatory state that can compromise the integrity of the vascular wall [14–16]. This physiopathological substrate is consistent with our data, which showed significantly worse basal VEINES-Sym and VEINES-QOL scores in obese patients, showing a burden of symptoms and a more pronounced impairment of quality of life in this group.

The high prevalence of more advanced CEAP classifications (C3-C6) in our total sample is not surprising, since the presence of complications such as edema, trophic skin changes, and ulcers is one of the main indications for invasive surgical treatment [18,19]. The fact that we did not observe a difference in the distribution of CEAP between the CEEE and EVLA groups suggests that, by this parameter, the severity of the disease was comparable, which strengthens the validity of the comparison of functional outcomes (quality of life and symptoms) between the two techniques.

Our results showed significant improvement in VEINES-Sym and VEINES-QOL scores and are aligned with studies that demonstrate the benefit of surgery. However, it is important to contextualize that this is not the unanimity in the literature. In the study by Rocha (2020) 20, which evaluated 92 patients, it was concluded that there was no difference in quality of life before and after surgery in most patients, which contrasts with our findings. This divergence can be attributed to methodological differences, such as the profile of the sample (in this study, 57.6% were CEAP 2, which suggests a population with less severe disease) or to the assessment instrument used. Our sample, with a significant proportion of patients in CEAP 3–6, may have presented greater scope for noticeable clinical improvement, reinforcing the idea that the benefit of surgery may be more evident in populations with more symptomatic or advanced disease.

Our results confirm that both EVLA and CEEE are effective therapeutic modalities for venous insufficiency, with statistically significant improvements in all strata of nutritional status (Table 2). This finding corroborates previous studies that also documented a substantial improvement in quality of life after interventional treatment for venous disease [20,21].

A particularly relevant aspect concerns the influence of nutritional status on postoperative outcomes. Our data revealed that patients with obesity had significantly worse baseline scores for VEINES-Sym and VEINES-QOL (Table 3), reflecting the additional impact of obesity on the severity of venous symptoms. However, the improvement observed after surgery was so expressive in this group that pre-existing differences between nutritional states were eliminated postoperatively. This result is in perfect harmony with a large VINES (2013) 22 study that evaluated 1445 patients and found a close relationship between body weight and clinical severity of primary venous disease, in which both overweight and obesity appear to be independent risk factors for increased severity. The mechanisms of this association go beyond the simple increase in hydrostatic pressure. The same study demonstrated that obese patients had more frequent axial reflux, incompetent perforating veins, and a critically higher prevalence of concomitant primary deep insufficiency. This positions obesity not only as an aggravating factor, but also as a modifier of the hemodynamic profile of CVD, making it structurally more complex [22–24].

However, the most enlightening outcome of our research was that, after surgery, the pre-existing differences between nutritional states were completely abolished in the postoperative. This finding is crucial and is supported in modern literature. A large study by Zottola (2024) [23]concluded that obese patients can obtain significant relief after treatment and, notably, show greater improvement in their perceived symptoms. Our data expand this conclusion to the CEEE technique, consistently demonstrating that the presence of obesity is not only not a contraindication for surgical treatment, but also that these patients obtain an absolute equivalent benefit, normalizing their quality of life in relation to eutrophic.

The direct comparative analysis between the techniques (Table 4) revealed that both produced equivalent improvements in the domain of symptoms (VEINES-Sym). However, in the specific domain of quality of life (VEINES-QOL), the EVLA group presented a significantly higher mean postoperative score. This difference, already documented in part of the literature, can be attributed to technical factors, such as concomitant sclerotherapy of tributary varicose veins in the same surgical act, or a potentially more comfortable postoperative recovery [19, 20, 21, 25].

Our research has limitations that deserve consideration. The observational and non-randomized design limits causal inference, and the allocation by service context introduces selection bias. The follow-up period of 60 days is insufficient to evaluate the durability of the results and the recurrence rates. Additionally, the option to use the worst score among treated limbs may have underestimated the actual therapeutic benefit. The lack of standardization in the method of application of the questionnaires (interview versus self-filling) among the groups may have introduced a benchmarking bias. Finally, the recruitment in a single center of a specific region limits the generalization of the findings to other Brazilian realities.

The choice between EVLA and CEEE should consider individual factors such as venous anatomical characteristics, comorbidities, availability of resources, socioeconomic context, and patient preferences. Both techniques represent important advances in the minimally invasive therapeutic arsenal for chronic venous disease.

## Conclusion

Our results reinforce the effectiveness of surgical techniques in improving the quality of life in CVD. They highlight obesity as a determinant of the worst preoperative quality of life, but more importantly, they validate surgical treatment as a high-benefit option for these patients, who can achieve a full and comparable postoperative quality of life. Nutritional status, therefore, should not be seen as a barrier, but rather as a factor to be considered for timely referral to treatment.

## Data Availability

The data that support the findings of this study are available from the corresponding author upon reasonable request.
